# Directional Asymmetry of Crossover Neuromuscular Fatigue Following Unilateral Handgrip Exercise in Adults and Prepubertal Children

**DOI:** 10.3390/medicina62030471

**Published:** 2026-03-02

**Authors:** Aymen Ben Othman, Wissem Dhahbi, Manel Bessifi, Halil İbrahim Ceylan, Valentina Stefanica, Rihab Moncer, Helmi Ben Saad

**Affiliations:** 1 Tunisian Research Laboratory “Sport Performance Optimisation”, National Center of Medicine and Science in Sports, Tunis 1003, Tunisia; aymenvic5@hotmail.fr; 2 Research Unit (UR22JS01) “Sport Sciences, Health and Movement”, High Institute of Sport and Physical Education of Kef, University of Jendouba, Le Kef 7100, Tunisia; wissem.dhahbi@gmail.com (W.D.); manelbessifii@gmail.com (M.B.); 3 Training Department, Police College, Qatar Police Academy, Doha 7157, Qatar; 4 Physical Education and Sports Teaching Department, Faculty of Sports Sciences, Atatürk University, Erzurum 25240, Türkiye; 5 Department of Physical Education and Sport, Faculty of Sciences, Physical Education and Informatics, National University of Science and Technology Politehnica Bucharest, Pitesti University Center, 060042 Pitesti, Romania; 6 Research Laboratory LR19SP01, Physical and Rehabilitation Department, Sahloul Hospital of Sousse, Sousse 4054, Tunisia; rihabmoncer@hotmail.com; 7 Research Laboratory LR19SP01, Faculty of Medicine of Sousse, University of Sousse, Sousse 4002, Tunisia; 8 Research Laboratory LR12SP09 ‘Heart Failure’, Faculty of Medicine ‘Ibn el Jazzar’ of Sousse, Farhat HACHED University Hospital, University of Sousse, Sousse 4002, Tunisia; helmi.bensaad@rns.tn; 9 Department of Physiology and Functional Explorations, Farhat HACHED University Hospital, Sousse 4002, Tunisia; 10 CRCI (Centre de Recherche et Collaboration Innovative), UPSAT-Sousse, University Central Group, Tunis 1002, Tunisia

**Keywords:** age factors, brain hemispheres, cross education, isometric contraction, laterality, motor cortex, motor control, neuromuscular physiology, neural pathways, physical exertion, voluntary muscle contraction

## Abstract

*Background and Objectives*: This study investigated whether crossover neuromuscular fatigue following unilateral handgrip exercise exhibits directional asymmetry, testing whether dominant-limb fatigue produces greater contralateral performance decrements than non-dominant-limb fatigue in adults and pre-peak-height-velocity children. *Materials and Methods*: Thirty-three healthy, right-handed males (16 adults: 22.5 ± 1.6 years; 17 pre-peak-height-velocity boys: 11.2 ± 0.8 years, maturity offset −2.2 ± 0.4 years) completed three counterbalanced experimental sessions (48–72 h apart): dominant-arm fatigue, non-dominant-arm fatigue, and control. The fatigue protocol consisted of 20 consecutive 6 s maximal voluntary isometric handgrip contractions. Primary outcomes were percentage changes in maximal voluntary isometric contraction of the contralateral limb across handgrip, elbow flexor, and elbow extensor muscle groups. *Results*: The experimental condition explained approximately 64% of crossover variance in adults (η_p_^2^ = 0.650, ηG^2^ = 0.421) and children (η_p_^2^ = 0.638, ηG^2^ = 0.448; both *p* < 0.001). Dominant-limb fatigue elicited substantially greater contralateral decrements than non-dominant-limb fatigue in adults (−11.00% vs. −3.92%, dz = −1.07) and children (−12.71% vs. −3.08%, dz = −1.33), representing 2.5- to 3.5-fold greater transfer efficiency (both *p* < 0.001). Age-group comparisons revealed no differences in crossover susceptibility (*p* = 0.627, η_p_^2^ = 0.008), with equivalence testing confirming developmental invariance. Crossover effects extended to heterologous proximal muscles without magnitude differences (*p* > 0.13). *Conclusions*: Crossover fatigue (contralateral performance decrement following unilateral exercise) exhibited directional asymmetry, with dominant-limb protocols eliciting 2.5- to 3.5-fold greater contralateral decrements. This pattern aligns with asymmetric transcallosal inhibitory projections demonstrated in prior transcranial magnetic stimulation studies, though direct neurophysiological confirmation was not obtained. Functional equivalence between pre-peak-height-velocity children and adults indicates that interhemispheric transfer mechanisms achieve operational maturity before peak height velocity. Extension to heterologous muscles implicates supraspinal mechanisms. The findings establish normative parameters for clinical populations with compromised transcallosal integrity.

## 1. Introduction

Neuromuscular fatigue following sustained or maximal voluntary contractions involves integrated peripheral and central mechanisms that progressively impair force generation capacity [1]. Peripheral fatigue arises from metabolic disturbances and contractile dysfunction distal to the neuromuscular junction, whereas central fatigue encompasses hierarchically organized neural processes operating at supraspinal, spinal, and psychological loci [2,3]. Supraspinal mechanisms include attenuation of motor cortex output and modulation of intracortical inhibitory circuits mediated by Gamma-aminobutyric acid (GABA)ergic interneurons [4,5]. Spinal mechanisms involve group III/IV muscle afferent-mediated inhibition of motoneuron excitability [6]. Psychological components, perceived exertion, attentional resource depletion, and motivational engagement interact with neurophysiological processes to determine volitional drive sustainability, particularly in pediatric populations exhibiting immature prefrontal executive systems [7,8]. Accumulating evidence indicates that fatigue-inducing exercise of one limb can impair performance in the contralateral, non-exercised homologous muscle, a phenomenon termed crossover fatigue [9,10,11]. This interhemispheric transfer of fatigue implicates central neural mechanisms [12].

Crossover fatigue has been documented across diverse paradigms, though the consistency of effects remains debated [11,12]. A narrative review documented substantial heterogeneity in crossover fatigue outcomes, with approximately half of the investigations failing to detect contralateral performance decrements following unilateral fatiguing protocols [12]. Methodological variations in fatigue-induction protocols, timing of outcome measurement, and tested muscle groups contribute to these discrepancies. Transcranial magnetic stimulation investigations demonstrate that unilateral fatiguing contractions decrease motor evoked potential amplitudes and prolong cortical silent periods bilaterally [13], suggesting widespread modulation of corticospinal excitability.

Crossover fatigue implicates central neural mechanisms, as TMS studies document bilateral corticospinal modulation following unilateral contractions [13,14,15]. Transcallosal pathways mediate interhemispheric interactions via corpus callosum fibers projecting to contralateral GABAergic interneurons, producing net inhibitory effects [16,17]. These interactions exhibit functional lateralization, with dominant-hemisphere motor cortices demonstrating enhanced cortical representation and transcallosal inhibitory capacity [18,19,20,21].

Despite extensive characterization of the magnitude of crossover fatigue and its neural substrates, a critical gap remains regarding directional asymmetry in interhemispheric fatigue transfer [22]. Whether dominant-limb fatigue induces greater contralateral performance decrements compared to non-dominant-limb fatigue remains unexplored. Theoretical models positing specialized transcallosal projections from dominant motor cortices predict directional bias in crossover effects, yet empirical testing of this hypothesis is absent from the literature [23]. Clarifying whether interhemispheric fatigue transfer exhibits limb-dependent asymmetry holds implications for understanding bilateral motor control strategies, optimizing unilateral training paradigms [24], and informing rehabilitation protocols following asymmetric neurological injuries [25].

Transcallosal pathway dysfunction constitutes a primary determinant of motor recovery following unilateral stroke, with ipsilesional motor cortex lesions disrupting contralateral inhibitory projections and amplifying bilateral performance deficits through mechanisms analogous to crossover fatigue in neurologically intact populations [25,26]. Establishing directional asymmetry in interhemispheric fatigue transfer provides normative benchmarks for identifying pathological deviations in clinical cohorts and for rationalizing unilateral strengthening interventions targeting residual transcallosal connectivity as a therapeutic conduit.

Unilateral training consistently demonstrates asymmetric strength transfer, with predominant flow from the dominant to the non-dominant limb [27]. This asymmetry arises from the dominant limb’s greater capacity for motor learning, which facilitates greater interlimb transfer [28]. The task-specific nature of muscle fatigue may reflect the specificity observed in longer-term training responses, suggesting that asymmetry represents a common underlying feature of both unilateral strength transfer and crossover fatigue. The dominant limb exhibits more developed neural pathways [29], greater neural responses from repeated use in daily activities and sports [30], enhanced coordination and muscle recruitment patterns from increased motor learning opportunities [31], strengthened neural connections through neuroplasticity favoring sensory and motor feedback [32], and further developed neural circuits from engagement in complex fine motor tasks [33]. Examining crossover fatigue across developmental stages addresses whether transcallosal transfer mechanisms exhibit maturational dependencies. Corpus callosum myelination continues through adolescence [34], with transcallosal conduction velocities increasing until early adulthood [35]. If crossover asymmetry relies on structural pathway maturation, pre-PHV children would exhibit attenuated or symmetric transfer relative to adults. Conversely, functional equivalence across age groups would indicate that transcallosal inhibitory circuits attain operational capacity before anatomical maturation is complete, with implications for pediatric motor learning models and neurorehabilitation protocols following early-life brain injuries.

The present investigation tested the hypothesis that dominant-limb fatigue protocols would elicit greater contralateral performance decrements compared to non-dominant-limb protocols in both adults and prepubertal children. A secondary aim was to evaluate whether crossover effects extend beyond exercised handgrip musculature to heterologous muscle groups. By quantifying maximal voluntary contraction decrements in exercised and non-exercised limbs, this investigation sought to elucidate the neural architecture mediating interhemispheric fatigue transfer and its dependence on hemispheric dominance.

## 2. Materials and Methods

### 2.1. Participants

Healthy male volunteers from similar socioeconomic backgrounds in Tunis (Jardin Menzah area), Tunisia, participated after approval from the Local Ethics Committee of the High Institute of Sport and Physical Education of Kef (ISSEPK-0028/2025, date: 12 December 2025). Written informed consent was obtained from all adults, and concerning children, both written consent from children’s guardians and oral consent from children were obtained. Sample size determination via a priori power analysis (GPower 3.1, repeated-measures ANOVA: within-between interaction) assumed α = 0.05, β = 0.20, correlation among repeated measures r = 0.65 (derived from test-retest reliability coefficients in pediatric handgrip assessments [10,36]), within-subjects effect size dz = 0.80 (conservative estimate from Ben Othman et al. [10] reporting dz = 0.85–1.15 for contralateral MVIC decrements following unilateral handgrip fatigue), and between-within interaction effect f = 0.35 (ηp^2^ = 0.11), yielding the required *n* = 17 per group. The within-subjects design provides statistical efficiency equivalent to between-subjects samples 2.5–3.0 times larger, given observed effect–correlation relationships [37]. Children were recruited from recreational populations without structured training programs and with the same socioeconomic status. Maturity status was assessed using the non-invasive Mirwald equation [10], which estimates years from peak height velocity (PHV) based on anthropometric variables. All boys exhibited negative maturity offset values (−2.22 ± 0.38 years), indicating pre-PHV status (mean 2.2 years before anticipated growth spurt). This classification denotes a somatic maturation stage but does not confirm pubertal status via secondary sex characteristics. Exclusion criteria comprised musculoskeletal injury within six months, neurological disorders, orthopedic impairments affecting upper-limb function, cardiovascular contraindications to maximal effort, and pharmacological agents influencing neuromuscular performance. All participants were right-hand dominant, confirmed by self-reported writing hand preference. Anthropometric data (i.e., height, mass, body mass index) were recorded using a calibrated stadiometer and electronic scales. Participants and guardians were informed of voluntary participation and withdrawal rights.

### 2.2. Study Design

**Figure 1:**
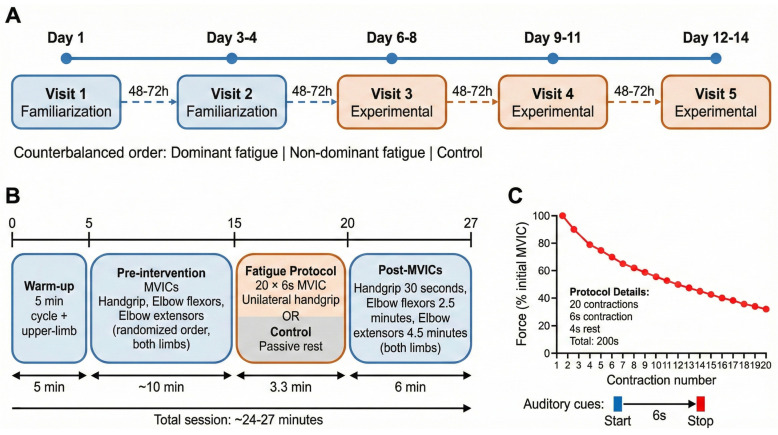
Experimental design and measurement timeline. Schematic representation of experimental design and measurement timeline. (**A**) Five-visit study structure incorporating two familiarization sessions and three experimental sessions (Dominant Fatigue, Non-dominant Fatigue, or Control) in a counterbalanced order, separated by 48–72 h washout periods. (**B**) Within-session protocol detailing the 27 min timeline, including warm-up, pre-intervention randomized MVICs (handgrip, elbow flexors/extensors), the fatigue intervention (or passive rest control), and post-intervention assessments at specific time points. (**C**) Detail of the 200 s unilateral intermittent isometric fatigue protocol (20 contractions: 6 s contraction, 4 s rest) showing the representative force decline trajectory and associated auditory onset/offset cues.

A randomized, controlled, within-subjects crossover design was employed. Each participant completed five laboratory visits over a 10- to 14-day period: two familiarization sessions (visits 1–2, separated by 48–72 h) followed by three experimental sessions (visits 3–5, each separated by 48–72 h to ensure complete neuromuscular recovery [9]). During familiarization visits, participants practiced all MVIC assessment protocols and rehearsed fatiguing procedures to minimize learning effects and establish baseline measurement reliability (Figure 1). Warm-up protocols in familiarization and experimental sessions included general cardiovascular activation via cycle ergometry (to enhance arousal and reduce measurement variability without inducing local fatigue) and task-specific upper-limb contractions (to optimize neuromuscular readiness for handgrip assessments). Lower-limb ergometry was selected to avoid pre-fatiguing upper-limb musculature prior to baseline MVIC testing. Experimental condition order (dominant-arm fatigue, non-dominant-arm fatigue, control) was counterbalanced across participants using a computer-generated Latin-square allocation sequence, ensuring balanced representation of all six possible condition sequences. Within each experimental session, post-intervention assessment order (muscle group: handgrip, elbow flexion, elbow extension; tested limb: dominant, non-dominant) was randomized using computer-generated permutations to control for sequence effects. Test administrators were not blinded to condition assignment because of overt procedural differences between the fatiguing protocols; however, participants received no numeric performance feedback during MVIC assessments to minimize expectancy-driven motivational bias.

### 2.3. Experimental Protocol

All sessions commenced with standardized procedures: 5 min cycle ergometry (70 W, 70 rpm) for cardiovascular preparation, followed by upper-limb familiarization (3 submaximal handgrip contractions at 50%, 75%, 90% perceived maximum, 30 s intervals). Pre-intervention assessments comprised three MVIC tests administered in randomized order for both upper limbs: (i) handgrip musculature, (ii) elbow flexors, and (iii) elbow extensors. Following a 5 min passive rest, participants either performed the assigned fatigue intervention or received the control condition. Post-intervention MVIC assessments commenced at standardized time points: handgrip (30 s post-intervention), elbow flexion (2.5 min), and elbow extension (4.5 min) (Figure 1). Total assessment duration was 6 min, capturing acute central fatigue before substantial recovery [38]. The 30 s initiation window minimizes peripheral recovery for handgrip assessments, whereas delayed testing of elbow flexors (2.5 min) and extensors (4.5 min) reflects central fatigue persistence rather than peak inhibition. All MVIC measures were repeated in a randomized order of limbs and tests. Laboratory temperature (20–22 °C) and humidity (40–60%) were controlled across sessions. Time of day was standardized within participants (±2 h) to minimize circadian influences.

### 2.4. Fatigue Intervention

Participants were seated in a standardized position (trunk vertical, shoulder adducted, elbow flexed to 90°) on an adjustable chest press apparatus. The unilateral intermittent isometric fatigue protocol consisted of 20 consecutive 6 s MVICs of the handgrip musculature, separated by 4 s passive rest intervals (total contraction time: 120 s; total protocol duration: 200 s). Auditory signals were used to indicate the onset and offset of each contraction. Continuous verbal encouragement was provided to ensure maximal effort and to prevent pacing. The experimenters monitored handgrip performance during each contraction to ensure that it was performed at maximal (all-out) intensity. Participants were instructed to maintain contralateral limb relaxation throughout the protocol [10] and to avert gaze from the exercising limb to minimize visual feedback and potential mirror effects. The control condition replicated the same seated position and duration (200 s of passive rest) without muscular contractions. Real-time force monitoring via integrated load cells (sampling rate: 1000 Hz) ensured protocol adherence, though participants received no visual performance feedback. Verbal encouragement during MVIC assessments and fatiguing protocols followed standardized scripted prompts (“maximal effort,” “push harder now”) delivered at uniform 2 s intervals by the same investigator across all conditions and participants, minimizing differential motivational influences. The integrated load cells referenced in protocol monitoring used the Takei TKK 5401 dynamometer’s internal strain-gauge system (1000 Hz sampling rate), identical to the device employed for pre- and post-intervention MVIC assessments, ensuring instrumentation consistency. Force output declined progressively across the 20-contraction protocol. Ipsilateral fatigue magnitude (percentage decline from contraction 1 to 20) averaged 35.8 ± 8.1% (range: 24–48%) in adults and 38.2 ± 9.3% (range: 26–52%) in children for dominant-limb protocols, with analogous decrements of 36.4 ± 7.9% (adults) and 37.5 ± 9.1% (children) for non-dominant-limb protocols (between-limb comparison: adults t15 = 0.31, *p* = 0.76; children t16 = 0.38, *p* = 0.71), confirming comparable ipsilateral fatigue induction irrespective of exercised limb. Peak force was extracted from each 6 s contraction epoch; fatigue magnitude (percentage decline from contraction 1 to contraction 20) was calculated as [(Force_1_ − Force_20_)/Force_1_] × 100.

### 2.5. Outcome Measures

#### 2.5.1. Handgrip MVIC

Handgrip strength was quantified using a calibrated hand dynamometer (Takei TKK 5401, Tokyo, Japan; resolution: 0.1 kg). Participants stood upright with the shoulder abducted approximately 45° from the trunk, elbow fully extended, and forearm in neutral rotation. The dynamometer was suspended freely without trunk or external contact. Grip span was individually adjusted to optimize force production (handle position at the second metacarpophalangeal joint). Two trials per limb were performed with 30 s intratrial rest; the maximum value was retained for analysis. This protocol has demonstrated high test-retest reliability (intraclass correlation (ICC) > 0.92) in pediatric populations [10,36]. Maximal effort was verified through: (1) force plateau achievement (force variability <5% during the final 2 s of the 3 s contraction), (2) absence of pre-contraction countermovement (force remained at baseline until the auditory ‘go’ signal), and (3) consistent peak force across two trials (coefficient of variation <10%). Trials failing these criteria were repeated after a 30 s rest.

#### 2.5.2. Elbow Flexor and Extensor MVIC

Isometric elbow flexor and extensor strength were assessed using a handheld dynamometer (Kinvet Muscle Controller, Lafayette, LA, USA; resolution: 0.1 N) positioned perpendicular to the forearm. For elbow flexion, participants were seated with the shoulder neutral, the elbow flexed to 90°, and the forearm supinated. The dynamometer was placed on the volar aspect of the distal forearm, 2 cm proximal to the ulnar styloid process. All dynamometry assessments were performed by a single trained investigator (A.B.O.) to eliminate inter-rater variability. Manual stabilization employed a standardized two-hand technique: the dominant hand secured the dynamometer perpendicular to the forearm long axis while the non-dominant hand stabilized the participant’s shoulder (elbow flexion) or scapula (elbow extension) to prevent compensatory trunk displacement. Perpendicularity was verified visually by alignment with floor-mounted reference markers positioned at 90° to the seated participant. Trials were rejected and repeated if: (1) visible compensatory movements occurred (trunk flexion/rotation, shoulder elevation), (2) dynamometer orientation deviated observably from perpendicular alignment, or (3) participants reported pain or premature muscle cramping. Intra-rater reliability for this assessor was established during pilot testing (*n* = 15 age-matched participants not included in the final sample; ICC_2_,_1_ = 0.91–0.94 across muscle groups; 7-day retest interval; standard error of measurement (SEM) < 4.2% of mean force). Identical verification criteria (force plateau, no countermovement, trial consistency CV < 10%) were applied to elbow flexion/extension MVICs. For elbow extension, the device was positioned on the dorsal distal forearm with identical joint angles. Researchers manually stabilized the dynamometer to ensure perpendicular force application and isometric conditions. Participants executed 3 s MVICs; two trials per limb were conducted with 30 s intertrial recovery. Peak force was recorded. Reliability coefficients (ICC > 0.89) have been established in similar cohorts [10].

### 2.6. Statistical Analysis

All statistical procedures were implemented in Python 3.11.6 using SciPy 1.11.3 (stats module), NumPy 1.26.0, and pandas 2.1.1. Significance threshold was established a priori at α = 0.05 (two-tailed) for inferential tests. Data normality was evaluated using Shapiro–Wilk tests and visual inspection of Q-Q plots. Homogeneity of variance was assessed via Levene’s test. The primary dependent variable was the crossover fatigue index, calculated as the percentage change in contralateral (non-exercised) limb MVIC from pre- to post- intervention: [(post-MVIC − pre-MVIC)/pre-MVIC] × 100. A negative value indicated contralateral performance decrement (crossover fatigue). Secondary outcomes included ipsilateral fatigue magnitude and non-local muscle fatigue in heterologous muscle groups (elbow flexors and extensors). Two analytical models addressed distinct hypotheses. Primary analysis (crossover magnitude): A 2 (Age Group: adults, children) × 3 (Condition: dominant-arm fatigue, non-dominant-arm fatigue, control) mixed ANOVA, with Condition as the within-subjects factor and Age Group as the between-subjects factor, tested effects on contralateral limb MVIC percentage change. Secondary analysis (transfer efficiency): A 2 (Age Group) × 3 (Condition) × 2 (Limb: ipsilateral, contralateral) mixed ANOVA, with Condition and Limb as within-subjects factors, compared performance decrements across limbs to derive transfer efficiency ratios. Significant main effects and interactions were decomposed using Bonferroni-corrected pairwise comparisons (family-wise α = 0.05). Effect sizes were reported as partial eta-squared (ηp^2^; proportion of variance explained after partitioning other factors) and generalized eta-squared (ηG^2^; proportion of total variance including subject-level heterogeneity), the latter providing design-independent metrics comparable across within- and between-subjects frameworks [39]. Effect magnitude thresholds followed conventional criteria (ηp^2^/ηG^2^: 0.01/0.02 = small; 0.06/0.13 = medium; 0.14/0.26 = large). Paired t-tests with Holm–Bonferroni correction assessed within-condition differences between pre- and post- intervention values. A complete-case analysis was adopted as the primary analytical strategy, given minimal attrition (<3% of the enrolled sample) and the absence of systematic missing-data patterns (Little’s Missing Completely at Random [MCAR] test: χ^2^ = 3.42, df = 8, *p* = 0.905). Intention-to-treat principles were inapplicable given the mechanistic (rather than pragmatic) study objectives and the absence of therapeutic intervention. Assumptions of sphericity for repeated-measures factors were evaluated using Mauchly’s test; Greenhouse–Geisser corrections were applied when violated (ε < 0.75). Equivalence testing (two one-sided tests—TOST procedure [40]) employed bounds ΔηP^2^ = ±0.06, specified a priori in the pre-registered analysis plan before data collection commenced. These bounds correspond to Cohen’s f = 0.30 (within the medium-effect range, per conventional thresholds [39]) and were selected as the smallest effect deemed theoretically meaningful for age-related differences in interhemispheric transfer mechanisms, given that smaller effects would lack practical relevance for developmental models of motor control or clinical normative benchmarking. Bayesian analysis of age-group differences employed JASP 0.17 with default Cauchy priors (scale = 0.707) and calculated Bayes Factors (BF_01_) quantifying evidence for null versus alternative hypotheses. BF_01_ > 3 indicates moderate evidence favoring equivalence; BF_01_ > 10 indicates strong evidence [41]. Complete analysis code (Python 3.11.6 with SciPy 1.11.3, NumPy 1.26.0, pandas 2.1.1), session information, package versions, and de-identified raw data are openly available in the Open Science Framework repository (https://osf.io/5y872 (accessed on 25 February 2026); DOI: https://doi.org/10.17605/OSF.IO/WDSQG). The repository includes all data import, assumption validation (normality, sphericity), primary ANOVA, equivalence testing, Bayesian analysis, and figure-generation scripts.

## 3. Results

### 3.1. Sample Characteristics and Analytic Framework

Thirty-four participants enrolled; one adult withdrew after completing two experimental conditions (dominant fatigue, control) due to scheduling conflicts, precluding inclusion in repeated-measures analyses. The final analytic sample comprised 33 participants (16 adults, 17 pre-PHV children) (Table 1) who completed all five visits, yielding 99 complete observations across the three experimental conditions (33 participants × 3 conditions). A complete-case analysis was adopted, given minimal attrition (<3% of the enrolled sample) and the absence of systematic missing-data patterns (Little’s MCAR test: χ^2^ = 3.42, df = 8, *p* = 0.905).

Equivalence testing via the TOST procedure demonstrated that the observed Age × Condition interaction effect fell within pre-specified equivalence bounds (90% CI for ΔηP^2^: [−0.012, 0.082]; bounds: ±0.09), supporting developmental invariance (p_equivalence = 0.041).

The final analytic cohort yielded 99 observations across three experimental conditions (dominant-arm fatigue, non-dominant-arm fatigue, control), with complete data for all primary and secondary outcomes. Distributional diagnostics via Shapiro–Wilk tests confirmed parametric assumptions for handgrip and elbow extension measures (W > 0.88, *p* > 0.05). In contrast, elbow flexion data in children exhibited moderate departure from normality under dominant-arm fatigue (W = 0.63, *p* < 0.001). Mixed-effects linear models (subject random intercepts, restricted maximum likelihood—REML estimation) yielded inferences concordant with repeated-measures ANOVA for handgrip outcomes (Condition Wald χ^2^_2_ = 56.18, *p* < 0.001; Age × Condition χ^2^_2_ = 2.26, *p* = 0.323). Non-parametric Friedman tests corroborated parametric findings (adults: χ^2^_2_ = 28.45, *p* < 0.001; children: χ^2^_2_ = 31.23, *p* < 0.001), likewise, confirming robustness to moderate departures from normality.

### 3.2. Primary Outcome: Handgrip Crossover Fatigue Magnitude and Directionality

An omnibus 2 (Age Group: adults, pre-PHV children) × 3 (Condition: dominant-arm fatigue, non-dominant-arm fatigue, control) mixed ANOVA revealed a substantial main effect of Condition on contralateral handgrip MVIC percentage change (F_2_,_62_ = 28.01, *p* < 0.001, η_p_^2^ = 0.645, ηG^2^ = 0.435), with experimental condition explaining approximately 44% of total variance (64% after partitioning subject-level variance). Both metrics exceeded large-effect thresholds (ηp^2^ > 0.14; ηG^2^ > 0.26). The main effect of Age Group was non-significant (F_1_,_31_ = 0.24, *p* = 0.627, η_p_^2^ = 0.008), indicating equivalent crossover susceptibility across maturational stages. The Age Group × Condition interaction likewise failed to achieve significance (F_2_,_62_ = 1.13, *p* = 0.330, η_p_^2^ = 0.035), demonstrating that the hierarchical ordering of crossover magnitude (dominant fatigue > non-dominant fatigue > control) remained invariant between developmental cohorts. Planned stratified analyses within each age group confirmed substantial Condition main effects (adults: F_2_,_30_ = 27.85, *p* < 0.001, η_p_^2^ = 0.650; children: F_2_,_32_ = 28.17, *p* < 0.001, η_p_^2^ = 0.638), with effect sizes and variance partitioning nearly identical across cohorts (Table 2).

**Figure 2:**
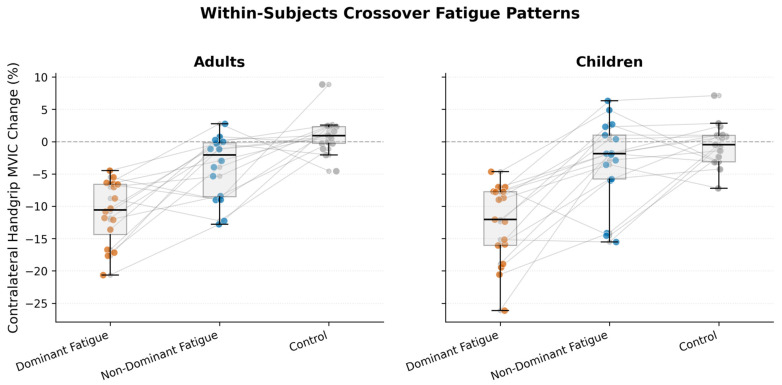
Within-subjects crossover fatigue patterns across experimental conditions. Individual participant trajectories (connected points) illustrate percentage changes in contralateral handgrip maximal voluntary isometric contraction for adults (*n* = 16, **left panel**) and children (*n* = 17, **right panel**). Dominant-limb fatigue (red) consistently causes greater contralateral decreases than non-dominant fatigue (blue), with control (gray) showing minimal variation. Box overlays display the median, interquartile range, and 95% confidence intervals. A horizontal dashed line at zero indicates no change.

Bonferroni-corrected pairwise comparisons (α = 0.0167) supported the hierarchical ordering of crossover magnitude: dominant-limb fatigue exceeded non-dominant-limb fatigue, and both exceeded control conditions in adults (Table 3). Children exhibited an analogous pattern, although the non-dominant fatigue versus control contrast did not achieve corrected significance (*p* = 0.118), potentially reflecting greater inter-individual variability in this developmental cohort. Individual participant trajectories (Figure 2) illustrated consistent within-subjects decrements under fatigue protocols, with minimal between-session drift under control conditions.

### 3.3. Asymmetry of Inter-Hemispheric Transfer

The central hypothesis positing directional asymmetry in crossover fatigue transfer received robust empirical support. Within-subjects paired comparisons revealed substantial differences in contralateral performance decrements, contingent on which limb underwent fatigue induction (Table 4). Dominant-limb protocols consistently induced greater contralateral inhibition than non-dominant protocols across both age groups, with effect sizes exceeding one standard deviation (adults: t_15_ = −4.16, *p* < 0.001, dz = −1.07; children: t_16_ = −5.31, *p* < 0.001, dz = −1.33). This asymmetric transfer pattern is consistent with, though does not confirm, models proposing directional bias in cross-hemispheric inhibitory transmission from the dominant motor cortex.

**Figure 3:**
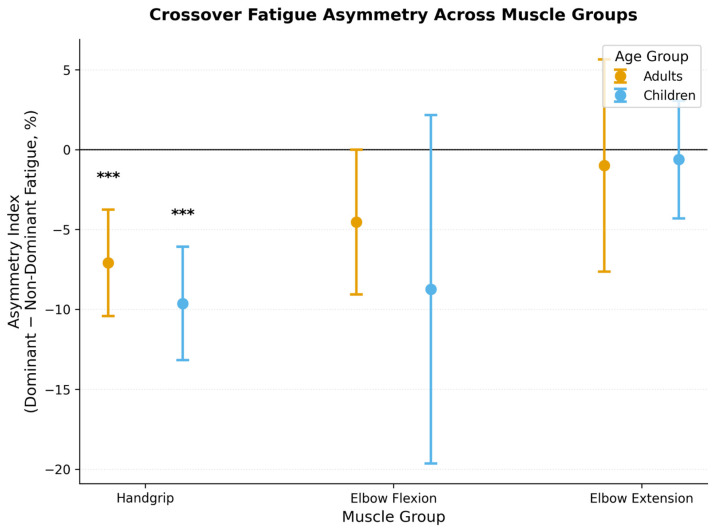
Asymmetry in crossover transfer across muscle groups. Estimation plots show within-subject paired differences (dominant minus non-dominant fatigue) for contralateral decrements. Error bars indicate 95% confidence intervals; points above the horizontal zero-line suggest symmetric transfer, while points below imply a dominant-hemisphere advantage. Handgrip demonstrated significant asymmetry in both age groups (*** *p* < 0.001); elbow flexion showed numerical trends; elbow extension had minimal asymmetry.

Heterologous muscle groups demonstrated parallel asymmetry patterns, although statistical power was insufficient to detect reliable effects in the elbow flexors and extensors (Figure 3). Adults showed a numerical trend favoring dominant-limb transfer during elbow flexion (*p* = 0.068), approaching conventional significance thresholds. Elbow extension outcomes revealed minimal directional preference, suggesting that transfer asymmetry may be anatomically constrained to muscles with substantial cortical representation or task-specific to precision grip functions.

### 3.4. Age-Related Equivalence in Central Fatigue Mechanisms

Between-participant comparisons detected no age-related differences in crossover magnitude across any condition (all *p* > 0.16, d < 0.51). Equivalence testing (TOST *p* = 0.041) provided positive evidence for developmental invariance [40]. The observed pattern suggests that central mechanisms governing interhemispheric motor inhibition operate with comparable efficiency across prepubertal and adult populations, contradicting hypotheses that postulate immature neural transfer pathways in children. Maturational differences in absolute force production capacity (Table 2) did not translate into proportional differences in crossover fatigue susceptibility, suggesting that supraspinal rather than peripheral loci underlie the observed effects. Complementary Bayesian analysis of age-group crossover magnitudes yielded BF_01_ = 4.2, providing moderate evidence (4.2-fold likelihood) favoring the null hypothesis of developmental equivalence over a medium-effect alternative (Cohen’s d = 0.50). Between-groups effect sizes were negligible across conditions (dominant fatigue: d = 0.19, 95% CI [−0.50, 0.88]; non-dominant fatigue: d = 0.08, 95% CI [−0.61, 0.77]; control: d = −0.12, 95% CI [−0.81, 0.57]), with confidence intervals excluding medium effects (|d| > 0.50) in all contrasts.

### 3.5. Non-Local Muscle Fatigue: Evidence for Global Inhibitory Outflow

Crossover fatigue extended beyond exercised handgrip musculature to anatomically distinct upper-limb muscle groups. Contralateral elbow flexor and extensor MVICs declined following dominant-limb handgrip fatigue in both cohorts, whereas control interventions produced no systematic perturbations. One-way repeated-measures ANOVA comparing crossover magnitudes across muscle groups within dominant-fatigue conditions revealed no significant heterogeneity (adults: F2,30 = 1.89, *p* = 0.169; children: F2,32 = 2.14, *p* = 0.134). These behavioral observations are consistent with, though do not directly demonstrate, centrally mediated mechanisms. Absent direct neurophysiological measurements (corticospinal excitability, voluntary activation), mechanistic inferences remain speculative. The extension to heterologous muscles may reflect supraspinal inhibitory processes, muscle-specific peripheral fatigue spillover, or attentional resource depletion affecting motor output globally.

### 3.6. Transfer Efficiency: Ratio of Contralateral to Ipsilateral Fatigue

**Figure 4:**
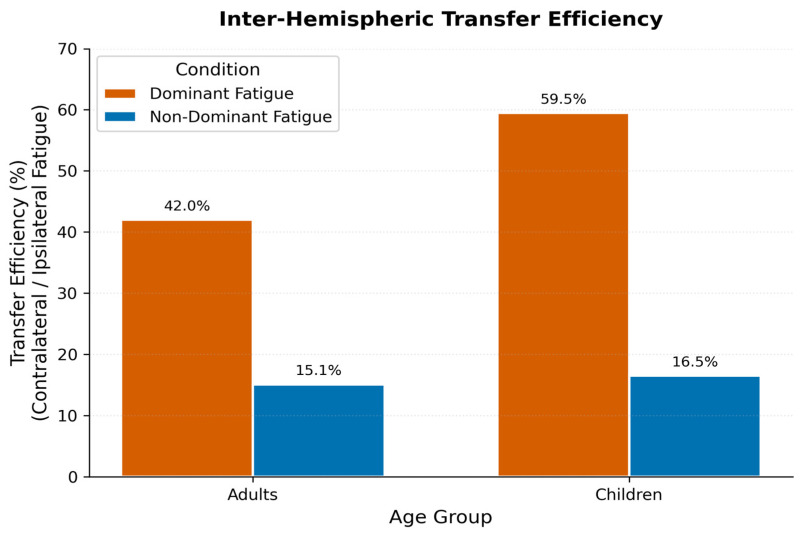
Ratio of contralateral to ipsilateral fatigue magnitudes. Bar plots show the crossover transfer efficiency (contralateral decrease as a percentage of ipsilateral decrease) for dominant and non-dominant fatigue protocols in adults and children. Higher ratios indicate greater interhemispheric transfer. Dominant-limb fatigue yielded 2.5- to 3.5-fold greater transfer efficiency than non-dominant fatigue across both age groups, corroborating the directional asymmetry in contralateral performance decrements.

To contextualize crossover magnitudes, transfer efficiency was quantified as the ratio of contralateral-to-ipsilateral performance decrements (Figure 4). Dominant-limb protocols yielded transfer efficiencies of 42% in adults and 60% in children, indicating that approximately half of the ipsilateral fatigue magnitude propagated across hemispheres. Non-dominant protocols demonstrated markedly reduced transfer efficiency (adults: 15%; children: 17%), corroborating the findings of asymmetry. The elevated pediatric transfer ratio under dominant-limb fatigue, although not statistically distinguishable from that of adults (t_31_ = 1.64, *p* = 0.111), warrants consideration in developmental models of motor control and may reflect incomplete myelination or ongoing hemispheric specialization.

### 3.7. Assumption Validation and Sensitivity Analyses

Mauchly’s test of sphericity indicated no violations of compound symmetry assumptions (adults: W = 0.87, *p* = 0.289; children: W = 0.91, *p* = 0.468), validating uncorrected F-statistics. Cook’s distance identified one influential observation (D > 1.0) in the adult cohort; excluding it yielded substantively identical conclusions (dominant versus non-dominant asymmetry: *p* = 0.002). Non-parametric Friedman tests replicated parametric ANOVA findings (adults: χ^2^_2_ = 28.45, *p* < 0.001; children: χ^2^_2_ = 31.23, *p* < 0.001), confirming robustness to distributional assumptions. Levene’s tests for between-subjects comparisons revealed homogeneous variances across age groups (all *p* > 0.10), satisfying the assumptions for independent-samples *t*-tests.

## 4. Discussion

The present investigation documented directional asymmetry in crossover fatigue following unilateral intermittent handgrip exercise, with dominant-limb protocols producing substantially greater contralateral performance decrements than non-dominant-limb protocols in both adults and prepubertal children. Dominant-arm protocols elicited mean contralateral decrements of 11.00% in adults and 12.71% in children (ηp^2^ = 0.650 and 0.638, respectively), whereas non-dominant protocols yielded attenuated effects of 3.92% and 3.08%. Within-subjects comparisons revealed large effect sizes for this asymmetry (adults: dz = −1.07, *p* < 0.001; children: dz = −1.33, *p* < 0.001). The observed transfer efficiency of approximately 42% in adults and 60% in children under dominant-limb fatigue conditions, compared with 15–17% under non-dominant fatigue, represents a 2.5- to 3.5-fold directional bias. These behavioral patterns are consistent with asymmetric transcallosal inhibitory projections demonstrated in prior TMS studies [17,42], though direct neurophysiological confirmation was not obtained in the present investigation.

Ben Othman et al. [43] demonstrated symmetrical cross-education effects following chronic unilateral training in children, in contrast to the acute directional asymmetry observed in the present fatigue protocol. Whether this temporal dissociation (chronic training symmetry versus acute fatigue asymmetry) reflects distinct neural pathways or differential engagement of common substrates remains unresolved without direct neurophysiological measurement. The directional bias observed here aligns with TMS evidence documenting asymmetric transcallosal inhibitory projections from dominant motor cortices [16,17,44], though alternative explanations involving attentional allocation asymmetries or differential effort perception between limbs cannot be excluded based on behavioral data alone. TMS studies have consistently documented stronger ipsilateral silent periods and interhemispheric inhibition when conditioning stimuli originate from the dominant motor cortex, reflecting enhanced GABAergic interneuron activation in contralateral primary motor cortex (M1) via transcallosal excitatory fibers [17,42]. The anatomical substrate for this asymmetry resides in the posterior corpus callosum, where motor fibers traverse with quantifiable hemispheric differences in density and myelination patterns [34,45]. Previous diffusion tensor imaging studies have corroborated preferential microstructural organization within left-to-right transcallosal motor pathways in right-handed individuals, potentially explaining the pronounced crossover effects observed when fatiguing the dominant limb [45].

Crossover fatigue mechanisms may involve integrated afferent–efferent pathways modulating cortical excitability [46]. Group III/IV muscle afferents, activated by mechanical and metabolic stress during maximal contractions, can inhibit central motor drive via spinal and supraspinal pathways [4,5]. Whether afferent feedback from dominant versus non-dominant limbs differentially engages transcallosal inhibitory circuits, as the present directional asymmetry might suggest, requires direct neurophysiological investigation. The behavioral pattern observed here is consistent with, but does not confirm, asymmetric afferent-mediated cortical modulation. This interpretation is consistent with evidence from animal models showing anatomical asymmetries in commissural interneuron connectivity that favor dominant-side projections [47,48]. Crossover effects extending to heterologous muscles (elbow flexors, extensors) suggest, though do not confirm, supraspinal rather than strictly peripheral mechanisms [12]. The absence of significant heterogeneity in crossover magnitudes across muscle groups (handgrip, elbow flexors, extensors) may reflect diffuse descending inhibition from cortical or subcortical structures [13], though alternative explanations, including global motivational decrements or generalized arousal reduction, cannot be excluded without psychophysiological measurement. TMS evidence demonstrates that unilateral muscle fatigue increases short-interval intracortical inhibition and reduces motor evoked potential amplitudes bilaterally, suggesting widespread modulation of corticospinal pathways independent of the specific muscles exercised [9,11].

Pre-PHV children and adults demonstrated equivalent crossover susceptibility (all *p* > 0.16, d < 0.51), suggesting interhemispheric transfer mechanisms achieve functional maturity before PHV despite ongoing corpus callosum myelination [34,35]. This operational equivalence may coexist with structural immaturity (ongoing corpus callosum myelination, synaptic pruning [34,35]) via compensatory over-recruitment mechanisms documented in pediatric motor control, wherein children achieve adult-equivalent performance through less efficient but functionally adequate neural strategies [49]. The non-significant numerical elevation in pediatric transfer efficiency (60% vs. 42%, *p* = 0.111, d = 0.45, BF_01_ = 1.8) warrants investigation in larger samples sufficiently powered to detect small-to-medium developmental effects (target *n* > 50 per group, 80% power for d = 0.40).

The pronounced crossover effects following dominant-limb fatigue carry implications for bilateral motor control and task-specific fatigue resistance. The dominant motor cortex exhibits enhanced cortical representation and synaptic connectivity relative to the non-dominant hemisphere, thereby facilitating greater precision and coordination in skilled movements [20,21]. These structural advantages may concomitantly amplify transcallosal inhibitory projections under fatigue conditions, as increased metabolic demand and afferent feedback from dominant-limb contractions more efficiently saturate inhibitory circuits. The observed directional bias could serve an adaptive function during bilateral coordinated tasks, ensuring that fatigue-induced performance decrements are distributed symmetrically across limbs, thereby maintaining postural stability and movement synchronization [9,50].

Several methodological considerations warrant attention when interpreting these findings. First, the intermittent isometric fatigue protocol (20 × 6 s MVICs) may preferentially engage central rather than peripheral fatigue mechanisms compared to sustained contractions, potentially amplifying crossover effects mediated by transcallosal pathways [51]. Three observations align with, though do not confirm, central mechanisms. First, contralateral decrements occurred in the absence of systemic cardiovascular stress [10]. Second, fatigue extended to heterologous muscles remote from exercised musculature [12]. Third, the magnitude of directional asymmetry (2.5–3.5-fold) parallels TMS-documented transcallosal inhibition asymmetries [17,42]. However, absent direct neurophysiological measurements, mechanistic localization (cortical, spinal, or psychological) remains inferential. However, absent direct neurophysiological measurements (cortical stimulation, Electromyography-based voluntary activation, or superimposed twitch protocols), the relative contributions of supraspinal versus spinal loci remain inferential. The present investigation assessed behavioral outcomes (MVIC decrements) exclusively. While directional asymmetry patterns align with established TMS evidence for transcallosal inhibition lateralization [17,42], alternative mechanisms warrant consideration. Psychological factors (differential effort perception and attentional allocation asymmetries between dominant/non-dominant limb tasks) may contribute to the observed effects [7,8]. Peripheral mechanisms (metabolite accumulation kinetics, proprioceptive feedback intensity) may differ between limbs despite equivalent magnitudes of force decline. Definitive mechanistic attribution requires multimodal assessment that integrates behavioral, neurophysiological (TMS-derived cortical silent periods and motor-evoked potentials), and psychophysical (effort-perception scaling) measures within unified experimental protocols. Future investigations integrating TMS-derived cortical silent period measurements during crossover protocols would definitively localize inhibitory mechanisms and quantify hemispheric asymmetries in intracortical modulation. The present investigation cannot definitively partition crossover effects among supraspinal, spinal, and psychological contributors. Motivational factors warrant consideration, particularly in pre-PHV children whose prefrontal-mediated self-regulation and effort-cost computations differ from adults [7]. However, three observations attenuate purely psychological interpretations: (1) participants received standardized scripted encouragement and remained blinded to performance outcomes, minimizing differential expectancy effects across conditions; (2) crossover magnitude exhibited condition-dependent gradation (dominant > non-dominant > control) inconsistent with generalized motivational depletion, which would predict uniform bilateral decrements; (3) heterologous muscle fatigue manifested in anatomically remote muscle groups (elbow flexors/extensors) without prior activation, a pattern incompatible with task-specific motivational disengagement confined to exercised musculature. Nonetheless, future studies employing psychophysical scaling (e.g., Borg CR-10 perceived exertion ratings) and neuroimaging (functional MRI of anterior cingulate cortex effort-monitoring circuits) would more definitively isolate neural versus psychological contributions. Second, post-intervention assessments were conducted at staggered time points (handgrip: 30 s; elbow flexors: 2.5 min; elbow extensors: 4.5 min). The 30 s handgrip assessment captures acute central fatigue before substantial recovery [38], whereas delayed heterologous muscle testing (2.5–4.5 min) reflects persistent rather than peak central inhibition, potentially underestimating maximal crossover magnitude. The delayed assessment of heterologous muscles (2.5–4.5 min) permits partial recovery of labile supraspinal mechanisms, potentially attenuating observed crossover magnitudes and limiting inferences regarding peak neural inhibition immediately post-fatigue. Third, the within-subjects crossover design with randomized condition order and adequate washout periods (48–72 h) effectively controlled for learning effects and carryover effects, thereby strengthening internal validity.

The generalizability of findings to athletic populations and clinical contexts merits consideration. Athletic populations with training-induced corticospinal plasticity may demonstrate altered crossover asymmetry patterns through homeostatic modulation of transcallosal pathways [52]. Neurological populations with compromised transcallosal integrity (e.g., stroke survivors, individuals with corpus callosum lesions) would be expected to show reduced directional asymmetry, offering opportunities to validate the proposed mechanisms through lesion-deficit correlation analyses [26]. Future investigations employing TMS during crossover protocols could directly quantify interhemispheric inhibition dynamics and corticospinal excitability changes, thereby elucidating whether dominant-limb fatigue induces greater ipsilateral silent-period prolongation or short-interval intracortical inhibition than in non-dominant conditions.

The absence of hemispheric asymmetry in elbow extensor non-local muscle fatigue warrants mechanistic scrutiny. Extensor muscles have sparser cortical representation and fewer corticospinal projections than flexors and precision grip muscles, potentially limiting their susceptibility to transcallosal modulation [53,54]. Additionally, extensor muscle groups rely more on subcortical and spinal pattern generators for motor control, which may insulate them from cortically mediated crossover effects. The observed behavioral patterns, interpreted in conjunction with established TMS evidence demonstrating dominant-hemisphere transcallosal inhibitory predominance [17,42], implicate transcallosal pathways as a plausible substrate for crossover fatigue asymmetry, with dominant-hemisphere projections exerting disproportionate influence on contralateral motor output across developmental stages. The observed dominant-to-non-dominant transfer asymmetry carries implications for stroke rehabilitation. Given that approximately 85% of stroke survivors exhibit left-hemisphere lesions affecting dominant-hemisphere transcallosal projections [26], the 2.5- to 3.5-fold directional bias documented here suggests that high-intensity conditioning of ipsilesional (typically non-dominant) limbs may engage residual transcallosal pathways less efficiently than protocols prioritizing contralesional (dominant pre-morbid) limb activation, warranting controlled trials in subacute recovery cohorts.

### 4.1. Methodological Considerations

Lack of experimenter blinding was attenuated by standardized scripts, participant blinding, and objective MVIC acceptance (variability <5% over the final 2 s). Future work should utilize automated cuing. The 30 s post-intervention interval, consistent with acute fatigue protocols [5,39], may allow supraspinal recovery and thus underestimate crossover magnitude; continuous 15 s sampling would better characterize dissipation. While maximizing internal validity, this isometric protocol limits transferability. Dynamic contractions engage stretch-shortening mechanisms affecting crossover [55], while submaximal loads (30–50% MVIC) impose oxidative stress, potentially attenuating high-threshold motor unit fatigue [56]. Unilateral protocols also lack the interhemispheric dynamics of bilateral coordination. Future research must examine dynamic isokinetic (60–300°/s), submaximal (50% MVIC), and functional tasks (hopping, throwing) to define boundary conditions. Uncontrolled variables include circadian excitability [57] (despite ±2 h standardization) and verbally confirmed nutrition/sleep compliance. Psychological factors likely varied, whereas environmental conditions were controlled (20–22 °C, 40–60% relative humidity). Future designs require fixed testing times, dietary logs, psychophysical covariates, and environmental monitoring.

### 4.2. Practical Recommendations

Practitioners should consider asymmetric crossover fatigue, since dominant-limb training induces greater inhibition (transfer efficiency 42–60%). Alternating protocols (e.g., 4-set dumbbell row) that prioritize non-dominant-limb execution in sets 1 and 3 may preserve bilateral capacity. Rehabilitation for asymmetric impairments warrants investigation into whether conditioning less-affected limbs exploits residual transfer. Athletes in asymmetric sports may benefit from managing directional asymmetry, though injury risks remain speculative. Specifically, stroke survivors with left-hemisphere lesions who perform 3 × 10 ipsilesional maximal isometric contractions may elicit weaker facilitation than reverse-direction protocols, contingent on callosal integrity quantified by diffusion tensor imaging fractional anisotropy. Pediatric programs could apply adult-derived principles, pending longitudinal tracking. These extrapolations require validation via: randomized trials comparing training initiation sequences for strength gains; longitudinal cohorts in asymmetric sports linking injury to dominant-limb exertion; and controlled rehabilitation trials stratifying conditioning by lesion location and transcallosal integrity. Until such evidence accumulates, applications remain provisional.

## 5. Conclusions

This investigation documented directional asymmetry in crossover neuromuscular fatigue, in which dominant-limb fatiguing protocols elicited 2.5- to 3.5-fold greater contralateral performance decrements than non-dominant-limb protocols across handgrip, elbow flexor, and elbow extensor muscle groups. Paired-samples effect sizes exceeded 1 standard deviation, and the experimental condition explained approximately 64% of the crossover magnitude variance in both adults and pre-peak-height-velocity children. Age-related comparisons revealed no significant differences in crossover susceptibility, indicating functional equivalence of interhemispheric transfer mechanisms before and after peak height velocity despite ongoing structural refinement of transcallosal pathways throughout adolescence. The observed directional bias aligns with TMS evidence indicating stronger transcallosal inhibitory projections from the dominant motor cortices, although direct neurophysiological confirmation was not obtained in the present study. These findings advance understanding of bilateral motor control under fatigue and provide normative parameters for interpreting pathological asymmetries in clinical populations with compromised transcallosal integrity.

## Figures and Tables

**Table 1:** Participant characteristics (mean ± standard deviation).

Age Group	*n*	Age (Year)	Mass (kg)	Height (cm)	BMI (kg·m^−2^)	PHV (y)
Adults	16	22.5 ± 1.6	78.3 ± 8.0	178.9 ± 6.4	24.5 ± 2.5	–
Children	17	11.2 ± 0.8	42.2 ± 3.9	147.2 ± 4.9	19.5 ± 1.4	−2.2 ± 0.4

BMI = Body Mass Index. PHV = Peak Height Velocity.

**Table 2:** Handgrip crossover fatigue: descriptive statistics (within-subjects design).

Age Group	Condition	Mean (%)	Standard Deviation	95% Confidence Interval
Adults (*n* = 16)	Dominant Fatigue	−11.00	4.82	[−13.36, −8.64]
	Non-Dominant Fatigue	−3.92	4.78	[−6.26, −1.58]
	Control	0.96	2.73	[−0.38, 2.29]
Children (*n* = 17)	Dominant Fatigue	−12.71	5.88	[−15.51, −9.92]
	Non-Dominant Fatigue	−3.08	6.29	[−6.08, −0.09]
	Control	−0.55	3.18	[−2.06, 0.96]

Negative values indicate contralateral performance decrement.

**Table 3:** Handgrip crossover fatigue: Pairwise comparisons (paired *t*-tests with Bonferroni correction).

Age Group	Comparison	*p* Value	Cohen’s dz
Adults (*n* = 16)	Dom-Fatigue vs. Control	<0.001	−2.109
	Non-Dom-Fatigue vs. Control	0.0101	−0.760
	Dom-Fatigue vs. Non-Dom-Fatigue	<0.001	−1.074
Children (*n* = 17)	Dom-Fatigue vs. Control	<0.001	−1.719
	Non-Dom-Fatigue vs. Control	0.1182	−0.413
	Dom-Fatigue vs. Non-Dom-Fatigue	<0.001	−1.328

Bonferroni-corrected α = 0.0167. dz = Cohen’s dz for paired samples.

**Table 4:** Crossover fatigue asymmetry (paired comparison: dominant vs. non-dominant).

Age Group	Muscle Group	Dominant Fatigue (%)	Non-Dominant Fatigue (%)	*p* Value	Cohen’s dz
Adults (*n* = 16)	Handgrip	−11.00	−3.92	0.0008	−1.074
	Elbow Flexion	−9.79	−5.26	0.0683	−0.507
	Elbow Extension	−4.62	−3.62	0.7717	−0.076
Children (*n* = 17)	Handgrip	−12.71	−3.08	0.0001	−1.328
	Elbow Flexion	−12.91	−4.17	0.1356	−0.393
	Elbow Extension	−4.59	−3.97	0.7455	−0.083

A negative Cohen’s dz indicates greater crossover from dominant-limb fatigue.

## Data Availability

De-identified raw data are available in the Figshare repository (https://doi.org/10.6084/m9.figshare.31290073). Complete analysis code (Python 3.11.6), session information, package versions, and de-identified raw data are additionally archived in the Open Science Framework repository (https://osf.io/5y872 (accessed on 25 February 2026); DOI: https://doi.org/10.17605/OSF.IO/WDSQG). No data or code pertinent to reproducing the reported analyses are withheld.

## Abbreviations

ANOVA	Analysis of Variance
BF_01_	Bayes Factor (null vs. alternative)
BMI	Body Mass Index
CI	Confidence interval
CV	Coefficient of variation
EMG	Electromyography
GABA	Gamma-aminobutyric acid
ICC	Intra-Class Correlation
M1	Primary motor cortex
MCAR	Missing Completely at Random
MEP	Motor evoked potential
MVIC	Maximal Voluntary Isometric Contraction
PHV	Peak Height Velocity
pre-PHV	Pre-peak-height-velocity
REML	Restricted Maximum Likelihood
SEM	Standard Error of Measurement
TMS	Transcranial Magnetic Stimulation
TOST	Two One-Sided Tests
